# Visualizing Uncertainty to Promote Clinicians’ Understanding of Measurement Error

**DOI:** 10.1177/10731911221147042

**Published:** 2023-02-01

**Authors:** Niek Frans, Benjamin Hummelen, Casper J. Albers, Muirne C.S. Paap

**Affiliations:** 1 Division of Mental Health and Addiction, Oslo University Hospital, Oslo, Norway; 2 Faculty of Behavioural and Social Sciences, University of Groningen, Groningen, The Netherlands

**Keywords:** visualization, communication, test score reports, measurement error, uncertainty

## Abstract

Measurement error is an inherent part of any test score. This uncertainty is generally communicated in ways that can be difficult to understand for clinical practitioners. In this empirical study, we evaluate the impact of several communication formats on the interpretation of measurement accuracy and its influence on the decision-making process in clinical practice. We provided 230 clinical practitioners with score reports in five formats: textual, error bar, violin plot, diamond plot, and quantile dot plot. We found that quantile dot plots significantly increased accuracy in the assessment of measurement uncertainty compared with other formats. However, a direct relation between visualization format and decision quality could not be found. Although traditional confidence intervals and error bars were favored by many participants due to their familiarity, responses revealed several misconceptions that make the suitability of these formats for communicating uncertainty questionable. Our results indicate that new visualization formats can successfully reduce errors in interpretation.

Psychological measurement plays an important supporting role in clinical decision making, by informing clinicians’ decisions regarding treatment options, effectiveness, and duration (Jensen-Doss, 2015). While psychometrically sound standardized tests provide an estimate of the construct we are trying to measure, this estimate has a degree of uncertainty which differs widely across instruments and possibly populations, which needs to be taken into consideration to make an informed decision (Charter, 2003). Test manuals usually report reliability estimates that express the degree of uncertainty associated with test scores (Charter & Feldt, 2001b; Gregory, 2015), and generally supplement this information with standard errors that can be used to calculate an uncertainty interval around an individual test score (Charter & Feldt, 2001a). Examples include the Wechsler’s Intelligence Scale for Children (Wechsler, 2014), the Wechsler’s Adult Intelligence Scale (Wechsler, 2008), the Minnesota Multiphasic Personality Inventory (Ben-Porath & Tellegen, 2008), and the Child Behavior Checklist (Achenbach, 1991). Several studies show that such abstract metrics can be difficult to interpret (Charter & Feldt, 2002; Hildon et al., 2012; McManus, 2012; Plebani et al., 2018; Simpkin & Armstrong, 2019), even for people who have received rigorous training (Belia et al., 2005; Kalinowski et al., 2018). Consequently, information on measurement uncertainty is often not considered when interpreting test scores (Hambleton & Zenisky, 2013; Plebani et al., 2018), which may create a false sense of certainty and diminish trust in test outcomes, when repeated score estimates do not align (Simpkin & Armstrong, 2019). Moreover, insight regarding measurement accuracy may have practical implications for decision making (Hopster-den Otter et al., 2019). For example, if an important cutoff score lies within the uncertainty interval, an observant clinician may rightfully decide that the outcome does not provide sufficient evidence to support a treatment decision and that more information is needed. As such, it is important to consider score report formats that clearly and intuitively incorporate the accuracy of a test score.

A growing body of literature points to the potential positive effects of visualization on uncertainty understanding. For example, a systematic review by Garcia-Retamero and Cokely (2017) on risk communication in health care showed that the use of visual aids is strikingly beneficial for a diverse audience of test users, including patients, physicians, and highly educated individuals. A recent review by Heltne et al. (2023) similarly found that visualizing uncertainty can improve participants’ understanding compared with commonly used numerical formats (e.g., confidence intervals). Particularly, visualizations that helped to indicate the shape of the uncertainty distribution, such as histograms and violin plots (see Figure 1C), successfully ameliorated important misconceptions about the likelihood of measurement errors. However, some of the most commonly used formats to visualize measurement accuracy (i.e., error bars) are frequently associated with a wide range of interpretation errors, including reinforcing categorical reasoning about probabilities (Helske et al., 2021; Levontin et al., 2020; Padilla et al., 2022), and misinterpreting the probability of values within the error bar (Levontin et al., 2020; Newman & Scholl, 2012). The inherent interpretation problems with error bars have led researchers to consider a variety of alternative uncertainty visualizations. It is difficult, however, to determine an “ideal” format for clinical test scores based on current literature, as the vast majority of studies use student samples or nonspecific samples to evaluate the viability of different uncertainty visualizations (Levontin et al., 2020; Meloncon & Warner, 2017), while hardly any studies include clinical practitioners (Heltne et al., 2023).

**Figure 1:**
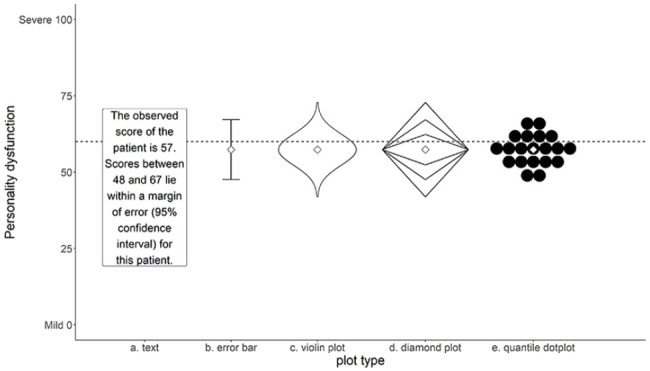
Five Formats Used in This Study: Text, Error Bar, Violin Plot, Diamond Plot, Quantile Dot Plot.

Padilla et al. (2022) make a broad distinction between two types of uncertainty visualization techniques: (a) graphical annotations that show properties of a distribution directly, including error bars, boxplots, and violin plots; and (b) mapping probability to visual encoding channels, such as color, blur, position, or transparency. Visualizations of the first type depict moments of a probability distribution and can give a representation of the uncertainty distribution of a score. Graphics of the second type have the advantage that they adjust a mark that is already in place, and as such do not require an additional spatial dimension. In one of the few studies concerning visualization of measurement error, Hopster-den Otter et al. (2019) showed that teachers participating in their study mostly found the visual encoding formats used in their study (i.e., blur and color value) confusing. Consequently, these formats either did not impact their decision process, or adversely affected their interpretation of test score uncertainty. Based on their findings, the authors recommended exploring other visualizations that incorporate a more direct representation of the probability distribution.

While studies generally demonstrate a positive influence of visualizations on the user’s interpretation of uncertainty, the literature on uncertainty communication so far shows that what “works” is highly dependent on (a) the type of uncertainty being depicted; (b) the type of judgment users need to make; and (c) characteristics of the user (Hullman et al., 2015; Levontin et al., 2020). Regarding the type of uncertainty, the systematic review by Heltne et al. (2023) shows that the number of studies that specifically evaluate the visualization of test score uncertainty can be counted on one hand. The same review indicates that different types of tasks require different types of visualizations. Generally, visualizations work best if the information that is needed to complete the task can be inferred directly from the visualization (Heltne et al., 2023). Furthermore, studies included in the review by Heltne et al. (2023) suggest that participants are sensitive to information overload and often made more optimal decisions when presented with simple, less detailed, visualizations. Notably, these studies generally presented participants with unfamiliar decision-making scenarios, which may have contributed to this conclusion. Presumably, clinicians’ expertise and training in score interpretation may reduce the risk of being overwhelmed by visualizations that are more complex, and allow clinicians to benefit from having more detailed information (Heltne et al., 2023). In this respect, visualizations that place less emphasis on confidence range limits might better represent the continuous nature of measurement uncertainty, and may help reduce categorical reasoning about uncertainty in users, while increasing accuracy (Correll & Gleicher, 2014; Helske et al., 2021). Many plot types (e.g., probability density function, boxplots, histograms, violin plots) use the surface area of a plot to represent uncertainty on a continuous scale. However, some researchers (e.g., Krider et al., 2001) suggest that estimating the surface area of a figure may be challenging for most people. Dividing the surface area into smaller meaningful areas or small countable quantities may aid the user’s interpretation (e.g., Kay et al., 2016).

Considering how characteristics such as education, graph reading ability (i.e., graph literacy), and statistical training have been shown to impact uncertainty understanding (Hopster-den Otter et al., 2019; Shah & Hoeffner, 2002; van der Bles et al., 2019; Zapata-Rivera et al., 2016), it is important to examine how to best communicate measurement accuracy in a population of clinical practitioners. This topic is particularly relevant, given that the abstract notions of standard errors and associated uncertainty intervals are repeatedly associated with interpretation problems in this population (McManus, 2012). Several studies suggest that there is ample room for improvement regarding the understanding of abstract statistical concepts by medical students and medical professionals (Hoffrage et al., 2000; Rutledge et al., 2004). Due to their vital role in test score interpretation, it is particularly important to explore formats that more accurately convey measurement accuracy in this population.

This study explores how different visualizations of uncertainty are related to clinicians’ understanding of measurement accuracy. We aim to provide specific recommendations for visualizing measurement uncertainty around test score estimates, to improve clinical professionals’ understanding. The results of this study may inform the development of score reports that present measurement accuracy in a user-friendly format.

## Method

### Design

A cross-sectional repeated measures design was used to compare different visual representations (see Figure 1) of measurement accuracy. Both traditional error bars (Figure 1B), representing the boundaries of a 95% confidence interval, and textual representations that report the limits of this interval (Figure 1A) were included as baseline conditions. Three additional visualizations were included based on recommendations by Padilla et al. (2022) and Heltne et al. (2023): a violin plot (Figure 1C), a diamond plot (Figure 1D) representing the familiar 68% and 95% confidence intervals, and a quantile dot plot (Figure 1E) with 20 dots sampled proportional to the quantiles of the distribution, so that each dot represents a 5% probability. Each visualization was designed to facilitate probability judgments in relation to a cutoff score, with the expectation that participants would (a) consider this probability when making a decision; and (b) relate their confidence about a decision to this probability. Uncertainty information was conveyed by the shape of the figures. Additional attributes (color, saturation, size, blur, etc.) were not manipulated, thus ensuring that observed differences in the results of this study were related only to differences in the composition of the visualizations. All visualizations included a visual representation of the estimated score.

### Population and Sample

The target population of this study consists of practitioners who are certified mental health providers in the fields of medical and behavioral sciences who deal with standardized test scores on a regular basis. A selective sample composed of Dutch and Norwegian psychiatrists, psychologists, remedial educationalists,^1^ and other professionals that are part of the target population was used in this study. Initially, participants were recruited from the authors’ professional network, after which snowball sampling was used by asking participants to extend the invitation to participate to others in their professional network that fit the target population. In addition, we approached several professional associations with the request to extend an invitation to their members. Both the Association of Educationalists in the Netherlands and the Norwegian Psychological Association cordially granted our request. Additional inclusion criteria were (a) working as a clinician in either Norway or the Netherlands; and (b) having current or past experiences with standardized score rapports. Based on a simulation study on sufficient sample sizes for multilevel modeling by Maas and Hox (2005), the target sample size was set at 100 participants from both countries. Sampling was terminated after 2 months, due to practical considerations, or sooner, if target sample size was met.

### Procedure

Ethical approval for this study was provided by the University of Groningen Pedagogical and Educational Sciences Ethical Committee. All visualizations were made with R version 4.1.1 (R Core Team, 2021) in the R package ggplot2 (version 3.3.2; Wickham, 2016) and presented in the same width and height ratio (4:3). All plot designs were pretested by two Dutch clinicians to ascertain whether the figures and textual explanations were understandable. Based on their feedback, a small adaptation was made to allow participants to go back and forth to the task explanation before viewing the graphs. Data were collected between March and May 2021.

Participants were informed about the goal of the study beforehand, and informed consent was obtained from each participant before starting the task. Each participant was shown five estimated scores with corresponding accuracy. Each score was randomly selected from the pairs shown in Table 1. These scores were presented once in each of the four visualization formats, and once in the textual format shown in Figure 1. Each visualization format included a cutoff score of 60 represented by a horizontal dotted line. The formats were presented in a random order, but always started or ended with the textual format to prevent participants from being forced to oscillate between visual and textual stimuli, which could be potentially confusing to them.

**Table 1.** Score and Cost Scenarios Used in This Study.

#	Score (*SE*)	*P* (score > 60)	Expected weeks waiting time for both departments					
			PD (5)	G (30)	PD (10)	G (30)	PD (15)	G (15)
1	48 (4)	.001	35	**30**	40	**30**	30	**15**
2	52 (4)	.023	34	**30**	39	**30**	30	**15**
3	56 (4)	.159	**30**	31	35	**32**	28	**17**
4	48 (8)	.067	33	**30**	38	**31**	29	**16**
5	52 (8)	.159	**30**	31	35	**32**	28	**17**
6	56 (8)	.309	**26**	32	**31**	33	25	**20**
7	48 (12)	.159	**30**	31	35	**32**	28	**17**
8	52 (12)	.252	**27**	31	**32**	33	26	**19**
9	56 (12)	.369	**24**	32	**29**	34	24	**21**

*Note*. Three observed scores and standard errors form nine score + *SE* combinations shown in the rows. The expected waiting time of three cost scenarios are shown in the last six columns. The correct decision (i.e., with the lowest expected waiting time) is printed in bold for each score and cost combination. PD = personality dysfunction.

The combinations of three observed scores and standard errors shown in Table 1 were chosen to ensure that (a) scores beyond the plot range (0–100) had a near-zero likelihood of occurring; (b) scores above the cutoff of 60 had varying probabilities ranging from almost zero (#1) to substantial (#9); (c) several combinations had probabilities near the visible boundaries of the visual formats (#1, #2, #3, #5, #7); and (d) some combinations had the same probability of overlap (#3, #5, #7).

Participants received a written instruction stating that each format showed the result of a screening test to measure the level of personality dysfunction (PD) of a patient. We chose to focus on PD due to its prevalence in clinical practice and the wide range of available standardized instruments (Tyrer et al., 2015). Their task was to refer this patient to a specialized department for personality disorders (Department PD) or a general outpatient clinic (Department G), based on the result of the screening test. It was recommended to refer patients with suspected true scores above the threshold score of 60 to Department PD. Participants were also provided a brief, one-sentence explanation of the features of each format (see Online Supplement).

After reading the task description and viewing the uncertainty format, participants were asked to make a probability assessment, by moving a slider between 0% and 100% to answer the question “What do you think is the probability that the patient’s actual score is above the cutoff point (i.e., higher than 60 as indicated by the dotted line)?^2^” Next, each participant was given a decision problem to either refer the patient to Department PD or Department G. Participants were told that the patient would be put on a waiting list and re-assessed after the waiting period. To increase generalizability of the results and avoid basing conclusions on one specific and somewhat arbitrary cost scenario, participants were randomly assigned to one of three scenarios, where the waiting time for Department PD ( ${\mathrm{wait}}_{PD}$ ) was 5, 10, or 15 weeks, and the waiting time for Department G ( ${\mathrm{wait}}_{G}$ ) was 30, 30, or 15 weeks, respectively. An incorrect initial referral would result in a combined waiting time for the patient. Table 1 shows the expected waiting time for both referral decisions in the 18 score and cost scenarios formed by the nine score and uncertainty combinations and the three different waiting times. Finally, participants were asked to rate their confidence in this decision on a 0 to 100 sliding scale, and to rank the five formats from most to least understandable.

### Measures

All measures were presented using an online Qualtrics questionnaire (qualtrics.com). Participants could choose between a Dutch or Norwegian version before starting the questionnaire.

#### Participant Characteristics

To differentiate among work contexts, participants were asked to indicate their profession and country of employment. Other participant characteristics, such as age and gender, were collected to acquire a more accurate sample description. Furthermore, participants’ research experience was assessed by asking whether they held a PhD, were a PhD candidate, or (co-)authored one or more published scientific articles. Extensive research experience may influence participants’ familiarity with standard errors, confidence intervals, and certain visualizations, which in turn may influence their performance (Shah & Hoeffner, 2002). Finally, since the size of the visualization may impact participants’ performance, the screen resolution for each participant was logged automatically.

#### Graph Literacy

The Subjective Graph Literacy scale (SGL; Garcia-Retamero et al., 2016) was administered to measure the participant’s graph literacy skills. This 10-item self-report questionnaire has shown high reliability in highly educated samples (α: .70–.89), and acceptable construct validity was suggested by high item-total correlations, and moderate correlations with other tests of graph literacy (Garcia-Retamero et al., 2016). In addition, the instrument has shown adequate predictive validity for interpreting graphical health risk information (Garcia-Retamero et al., 2016). Each item can be answered on a 6-point Likert-type scale, where higher scores indicate higher self-reported graph literacy. With permission of the SGL authors, all items were translated from English into Dutch and Norwegian by professional translators. A first translation from English into Dutch or Norwegian was done by a Dutch and a Norwegian native speaker, respectively, who were fluent in English. These translations were then back-translated into English by two different translators with similar proficiency to evaluate translation accuracy. Any deviations from the original translations were discussed with the first two translators, and corrections were made in the translations if required. The translated instruments showed good reliability ( $\lambda_{2}$ = .86), and exploratory and confirmatory Mokken scale analyses (Mokken, 1971) indicated that the items formed a unidimensional scale with adequate scalability (*H* = .42). Consequently, the total score was used to represent graph literacy skills which ranged from 10 to 60. There were only small differences in terms of psychometric properties between the Norwegian ( $\lambda_{2}$ = .90, *H* = .51) and Dutch ( $\lambda_{2}$ = .85, *H* = .38) translations.

#### Outcome Measures

The outcome measures of this study were (a) inaccuracy of probability assessments; (b) decision quality; (c) subjective confidence in decision; and (d) subjective understanding. The inaccuracy of participants’ probability assessments was defined as the difference ${\hat{P}}_{ij}-P_{i}$ between the probability ${\hat{P}}_{ij}$ specified by participant *j* on item *i* and the actual probability Pi of a score higher than 60 for the particular item *i* shown in Table 1. A positive inaccuracy indicates the degree of overestimation, while a negative inaccuracy indicates the degree of underestimation.

Decision quality was evaluated in terms of the expected waiting time for the fictional patient, by comparing the expected waiting time for the decision made by the participant, to the expected waiting time for the alternative. The expected waiting time was calculated from the probability of a score higher than 60 Pi and the waiting times for department PD ${\mathrm{wait}}_{PD}$ and department G ${\mathrm{wait}}_{G}$ . For department PD, the expected waiting time was defined as $P_{i}\times {\mathrm{wait}}_{PD}+(1-P_{i})\times ({\mathrm{wait}}_{PD}+{\mathrm{wait}}_{G})$ . For department G, the expected waiting time was defined as $\left(1-P_{i})\times {\mathrm{wait}}_{G}+P_{i}\times ({\mathrm{wait}}_{PD}+{\mathrm{wait}}_{G}\right)$ . A decision was marked as “correct” if the expected waiting time (shown in Table 1) for the selected department was lower than the expected waiting time for the alternative.

Subjective confidence and understanding were retrieved directly from the confidence indicated by the participant on a range of 0 to 100 and the understandability ranking assigned to each of the five formats. In addition, participants were asked to motivate their ranking in an open-ended question. These qualitative statements were included to obtain a better idea of specific visualization features that aided or hindered participants’ understanding.

### Analyses

All analyses were performed in R v4.1.3 (R Core Team, 2022). After a descriptive analysis of the sample characteristics, inaccuracy of probability assessments, and decision quality, mixed-effect beta regression models (Brooks et al., 2017; Ferrari & Cribari-Neto, 2004) were used to predict absolute assessment inaccuracy $I_{ij}=|{\hat{P}}_{ij}-P_{i}|$ across different formats. Beta regression is a flexible method that can handle bounded dependent variables in the interval (0,1) with non-normal distributions. Unlike alternative approaches (e.g., transforming the dependent variable), model parameters can be easily interpreted in terms of the original response. Due to these advantages, beta regression is commonly used for modeling outcomes such as proportions and rates (Ferrari & Cribari-Neto, 2004). Since these models assume an outcome that is larger than 0 and smaller than 1, absolute inaccuracy was compressed slightly using a transformation by Smithson and Verkuilen (2006): $I_{ij}^{\prime }=(|{\hat{P}}_{ij}-P_{i}|(N-1)+0.5)/N$ , where *N* indicates the sample size. A logit link function was used, which facilitates interpretation of regression parameters as an odds ratio (Ferrari & Cribari-Neto, 2004). All models were estimated using the glmmTMB package (Brooks et al., 2017) and take the following form:

$$logit\left(I_{ij}^{\prime }\right)\sim \beta_{0j}+\beta_{1,\ldots ,4}format_{ij}+\beta_{\ldots }characteristics_{j}+R_{ij}$$

$$\beta_{0j}\sim \gamma_{00}+U_{0j}.$$

Here, $U_{0j}$ described interindividual differences (i.e., random effects) in respect to the overall inaccuracy $\gamma_{00}$ of the probability assessments by participant *j*. The model assumes that these individual deviations can be expressed by a normal distribution with mean 0 and variance $\tau_{0}^{2}$ . The other β coefficients indicate the overall (fixed) effect of visualization formats on probability assessment inaccuracy as well as the effect of participant characteristics (i.e., graph literacy skills, profession, experience, and country of residence). Finally, unexplained intraindividual differences are represented by the error term $R_{ij}.$

Addition of fixed and random effects to the model was based on descriptive findings of the relation with probability assessment inaccuracy and improvement to model fit as assessed by the Akaike information criterion (AIC). Participant characteristics were retained in the model if they reduced the AIC of the model. Since decision quality was defined as a dichotomous correct/incorrect variable, we used a generalized multilevel model with decision quality as the dependent variable to analyze this outcome. Fixed and random effects were included in this model in the same manner as the inaccuracy model.

To explore whether participants expressed more confidence in their decision, when the difference in expected waiting time between the chosen optimal outcome and the alternative was larger, we took the difference in expected waiting time between the chosen outcome and the alternative, so that larger negative scores represent decisions that are more incorrect, and larger positive scores reflect decisions that are more correct. This outcome was correlated with the amount of confidence for each visualization format.

Finally, we explored differences in subjective understandability by evaluating the average participant rankings for different formats. A qualitative content analysis (Forman & Damschroder, 2008) was also conducted on participants’ textual statements concerning features that influenced understandability. All participants’ statements about understandability were open-coded in Excel by the first author (for the Dutch subsample) and a graduate student who is fluent in Norwegian (for the Norwegian sample). To explore format-specific features that aid or hinder understanding, participants’ statements were grouped by visualization format. Coded statements were reported when they were mentioned by at least two participants.

## Results

### Non-Response and Missing Data

Of the 335 participants who opened the questionnaire, 304 gave informed consent and continued to the questions. Only participants who viewed at least one uncertainty format (*n* = 239, 78.6%) were retained in the data set. The 65 participants who dropped out before this point did not differ markedly in regard to any of the included demographic characteristics; except that a more sizable proportion of these 65 participants (6.2%) never worked with standardized tests compared with participants who did view the visualizations (1.3%).

An additional number of participants were removed from the data set, because they worked in a country other than Norway or the Netherlands (*n* = 2), or worked in a non-clinical profession (*n* = 7). The final sample consisted of 230 participants. Most of these participants (*n* = 200, 86.9%) responded to all uncertainty formats. Of the 30 participants who dropped out before viewing all visualizations, 22 participants only viewed one format. More often than expected, dropout occurred after participants had viewed only the textual format or the error bar; the other formats were all underrepresented within the group that dropped out.

### Sample Demographics

Table 2 shows the sample demographics of the 230 participants split by country. The majority of participants (66.1%) worked in the Netherlands. Dutch participants were mostly women who worked as remedial educationalists. The Norwegian sample was slightly more evenly distributed regarding gender, and the majority worked as clinical psychologists. In both countries, the largest age group was between 30 and 39 years. However, Norwegian participants were generally older and worked with standardized assessment on a more regular basis. A large proportion of the sample consisted of participants who had a PhD degree or had (co-)authored a scientific study, and in that capacity had acquired some research experience. The Norwegian sample, especially, included a large number of participants with research experience.

**Table 2.** Sample Characteristics (%), Split by Country.

Variable	Netherlands *n* = 152	Norway *n* = 78	Total *N* = 230
Gender			
Female	92.8	61.5	82.2
Male	7.2	38.5	17.8
Profession			
Psychologist	28.3	92.3	50.0
Remedial educationalist	69.7	0.0	46.1
Psychiatrist	2.0	5.1	3.0
Psychiatric nurse	0.0	2.6	0.9
Age			
20–29	30.3	15.4	25.2
30–39	40.8	33.3	38.3
40–49	18.4	23.1	20.0
50–59	6.6	21.8	11.7
>60	3.9	6.4	4.8
Work with standardized tests			
Daily	11.2	21.8	14.8
Weekly	46.7	57.7	50.4
Monthly	30.9	12.8	24.8
Biannually	9.9	5.1	8.3
Annually	0.7	0.0	0.4
Never	0.7	2.6	1.3
PhD			
Yes	5.9	17.9	10.0
Not yet	2.6	17.9	7.8
No	91.4	64.1	82.2
Published author			
First author	9.2	34.6	17.8
Co-author only	3.9	20.5	9.6
Never	86.8	44.9	72.6
SGL score^a^			
Mean (*SD*)	38.3 (6.4)	40.0 (7.8)	38.8 (6.9)

a Subjective Graph Literacy scale (Garcia-Retamero et al., 2016).

### Inaccuracy of Participants’ Probability Assessments

When looking at the untransformed difference between participants’ probability assessments and the true probability underlying each score, inaccuracy of probability assessments was positively skewed with a median of .06 (median absolute deviation [MAD] = .10). When aggregating over participants, most participants tended to overestimate the probability of scoring above the cutoff, with a median average error of .08 (MAD = .09). Nine outliers could be identified. Seven participants made average overestimations between .41 to .74, and two underestimated by an average of –.19 and –.32. Although, these participants could not be distinguished by any specific characteristics, four of the nine outliers belonged to participants who did not finish the questionnaire. In fact, median average inaccuracy for participants who did not view all formats (*n* = 30, *Mdn* = .16) was more than twice as high compared with participants who did view all formats (*n* = 200, *Mdn* = .07). However, this difference was not significant (*W* = 2304.5, *p* = .13). There was no reason to assume that these outliers were invalid observations; they were therefore retained in further analyses.

**Figure 2.**
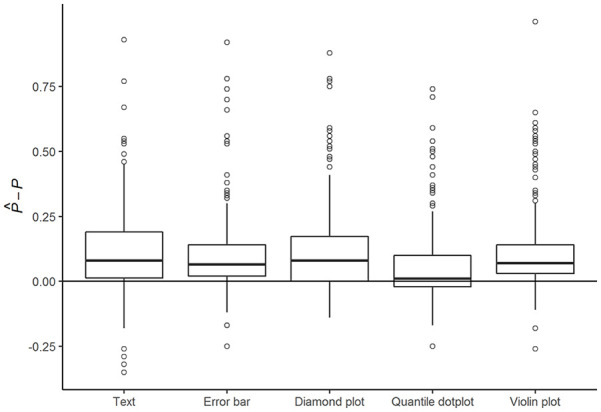
Inaccuracy of Probability Assessments $I_{ij}={\hat{P}}_{ij}-P_{i}$ by Format. *Note.* The horizontal line indicates a perfectly accurate probability assessment by the participant (i.e., ${\hat{P}}_{ij}=P_{i}$ ).

Figure 2 shows the distribution of participants’ probability assessment inaccuracy for each of the five formats. The vast majority overestimated the probability $P_{i}$ with a median overestimation of .06, regardless of the presentation format. Quantile dot plots led to the smallest median overestimation (*Mdn* =.01, MAD = .08). Error bars and violin plots performed second best (*Mdn* = .07, MAD = .09), followed by diamond plots and text (*Mdn* = .08, MAD = .12). The interindividual variation for probability assessments in the last two formats (diamond and text) seemed to be somewhat higher than the interindividual variation in the other three formats.

Table 3 shows the absolute inaccuracy of participants’ probability assessments for three different models: one model without explanatory variables (the empty model), one with only format as an explanatory variable, and finally the most complete model with all explanatory variables that improved model fit. The first model shows that average participants’ probability assessments significantly deviated from a perfectly accurate assessment of the probability of PD (*p* < .001). The Level 2 variance component shows that probability assessment inaccuracy varied considerably across participants. The assessments of participants whose inaccuracy was estimated as one standard deviation above average were estimated to be nearly twice as inaccurate as the assessment of the average participant. Inaccuracy of probability assessments across formats were moderately correlated within individual participants (*ICC* = .41).

**Table 3:** Parameter Estimates of a Multilevel Beta Regression With Absolute Inaccuracy of Participant Probability Assessments I’ij as the Dependent Variable.

Variable	Empty model	Format			Format + characteristics		
	Estimate (*SE*)	Estimate (*SE*)		*OR*	Estimate (*SE*)		*OR*
Intercept (text)	−1.96 (0.054)*	−1.81 (0.075)*			−1.72 (0.080)*		
Format							
Error bar		−0.15 (0.086)		0.86	−0.15 (0.086)		0.86
Diamond plot		−0.17 (0.086)*		0.84	−0.17 (0.086)*		0.84
Quantile dot plot		−0.43 (0.087)*		0.65	−0.43 (0.087)*		0.65
Violin plot		−0.09 (0.085)		0.92	−0.09 (0.085)		0.92
(Co)-Author					−0.32 (0.113)*		0.73
Level 2 variance τ2 $\left(\tau^{2}\right)$	0.389	0.399			0.380		
Precision parameter φ	7.10	7.37			7.37		
AIC	−2293.3	−2312.4			−2318.3		

*Note.* A value of 0 indicates perfect accuracy. All models were estimated on 1,039 responses of 228 participants. AIC = Akaike information criterion.

* *p* < .05.

While none of the formats completely eliminated bias in participants’ probability assessments, the second model shows that both diamond plots (*OR* = 0.84, *p* = .043) and quantile dot plots (*OR* = 0.65, *p* < .001) significantly improved assessment accuracy compared with textual formats. Post hoc comparisons further showed that the quantile dot plot led to significantly more accurate probability assessments than all of the other formats, with assessments that were 1.4 times more accurate compared with assessments based on violin plots (*p* <.001), and 1.3 times more accurate compared with diamond plots (*p* = .004) and error bars (*p =* .002).

The last model shows that participants with experience as a (co-)author were significantly more accurate overall compared with participants without authorship experience (*OR* = 1.4, *p* = .005). After accounting for authorship, none of the other characteristics (i.e., experience with standardized tests, having a PhD, subjective graph literacy, age, gender, profession, or country) were significantly related to difference in participants’ probability assessment inaccuracy. For that reason, coefficients for these characteristics were not included in the last model. Removal of participants with large standardized residuals or large random effects had no noticeable effect on the results; that is, none of the regression coefficients changed from being significant to not being significant or vice versa. The largest change in coefficient size was seen in the violin plot, which decreased by 0.016 when removing four observations with large residuals (>1.5), and by −0.05 when removing four participants with large random effects (>1.5).

### Decision Quality and Confidence

The majority of participants’ referral decisions (72.2%) were correct decisions, resulting in the lowest expected waiting time for the fictional patient. In accordance with participants overestimating the probability of PD, patients were referred to Department PD slightly more often than necessary (i.e., even when the *expected* waiting time was longer for this department; see Table 1). Around three quarters (73.1%) of the time, participants chose to refer to Department G; the majority of these decisions (77.3%) were correct. Of the remaining 26.9% referrals to Department PD, a large proportion (41.7%) was made despite the longer expected waiting time for this department. A sizable proportion of referrals to Department PD (31.9%) was made, even when the expected waiting time for the patient was more than a month longer compared with referring the same patient to Department G.

**Figure 3.**
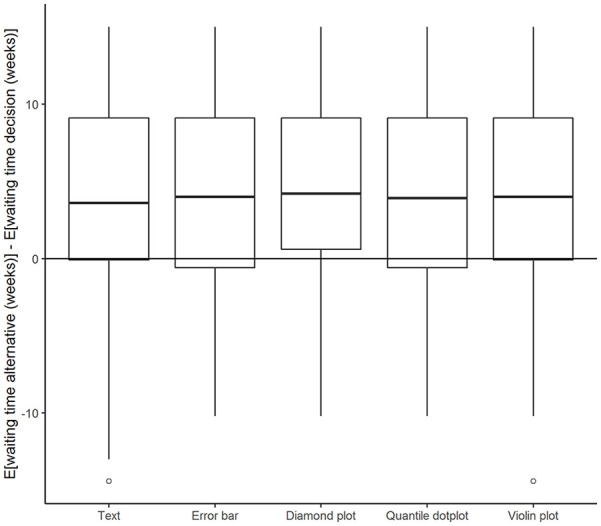
Difference in Expected Waiting Time for Decisions (Y-Axis) Plotted Against Different Formats. *Note.* Y-values lower than 0 indicate an incorrect decision.

Decision quality was moderately correlated with absolute inaccuracy (*r* = –.27). As expected, more accurate assessments of probability tended to be associated with higher quality decisions. However, as Figure 3 shows, there was no relation between format and decision quality. Counterintuitively, the quantile dot plot that was associated with more accurate probability assessments appeared to lead to worse decisions somewhat more frequently. However, these differences were small and non-significant. After accounting for differences in inaccuracy, there were no participant characteristics that significantly predicted decision quality. Results for the empty model, model inaccuracy, and format as independent variables can be found in the online supplement (Supplemental Table S1).

As expected, subjective confidence ratings were higher when the decision was easier; that is, when the difference in a chosen optimal outcome and the alternative expected outcome was larger (*r* = .22). However, there was no significant or relevant difference in the strength of this relation for different formats, or in the overall confidence level for different formats.

### Subjective Understandability

Participants in both countries were similar regarding their ratings of understandability. Table 4 shows the distribution and mean rank for each format. The classical formats (text and error bar) were rated as most understandable by 61% of participants, while diamond plots and violin plots received the lowest ranking. Although the quantile dot plot was deemed only slightly more understandable than diamond and violin plots, when looking at the mean rank, participants were more divided on this plot type compared with the other formats. One fifth of participants preferred the quantile dot plot over all other formats, but one in four rated this plot as least understandable. Unsurprisingly, participants who rated the textual format as most understandable scored significantly lower on subjective graph literacy, *F*(4, 188) = 8.0, *p* < .001. No other characteristics were related to perceived understandability.

**Table 4.** Proportion of Participants (n = 194) That Ranked Each Format From Most Understandable (1) to Least Understandable (5).

Understandable	Textual	Error bar	Diamond plot	Quantile dot plot	Violin plot
1 (Most)	.30	.31	.09	.20	.10
2	.22	.28	.18	.12	.20
3	.16	.16	.23	.25	.19
4	.12	.18	.26	.17	.27
5 (Least)	.19	.07	.24	.26	.24
Mean rank	2.7	2.4	3.4	3.2	3.3

Coding of the qualitative responses showed that familiarity played a major role in participants’ ranking of understandability. Participants frequently mentioned this reason for finding text (*n* = 14) and error bars (*n* = 22) understandable, and other formats less understandable (*n* = 16). Textual intervals were often seen as more concrete (*n* = 18), exact, and objective (*n* = 14). Although some participants indicated a preference for a textual format (*n* = 5), the lack of visual support (*n* = 18) and, in particular, a probability distribution (*n* = 6) were also frequently mentioned as downsides of the textual format. The fact that the probability distribution was not provided was similarly mentioned as a downside of the error bar format (*n* = 18). Moreover, two participants mentioned that the distribution was misrepresented by error bars. On the other hand, the concise format of the error bar (*n* = 6) was considered to provide a quick and understandable overview (*n* = 26).

By design, the violin plot, diamond plot, and quantile dot plot include more information than the textual or error bar formats, as they provide a more detailed graphical representation of the underlying probability distribution. Some participants were of the opinion that the three visualization formats more clearly showed the distribution (*n* = 20), while others deemed these formats to be too complicated (*n* = 12) or crowded (*n* = 19). Although participants indicated that the violin plot clearly showed the distribution (*n* = 18), some found it difficult to assess a probability (*n* = 8), interpret the width (*n* = 3), or make accurate probability assessments (*n* = 2) using this format. The width of the diamond plot was similarly mentioned as unclear (*n* = 2). Although some participants had a positive opinion about the multiple intervals provided in the diamond plot (*n* = 15), others found them difficult to interpret (*n* = 21). A particular downside mentioned for the quantile dot plot was that dots could fall directly on top of the cutoff line (*n* = 4), which occurred in all but one of the visualizations shown in this study, making interpretation more difficult. Participants in general were positive about the clearly visible percentage (*n* = 16) in the quantile dot plot that was made easy by counting (*n* = 4) and provided a clear representation of the probability above the cutoff (*n* = 9).

## Discussion

This study explored how different visualizations of uncertainty are related to clinicians’ understanding of measurement uncertainty. We evaluated Dutch and Norwegian clinicians’ task performance on inaccuracy of probability assessments, decision quality, and subjective understanding for five different score formats. Overall, clinicians in both countries were reasonably able to interpret the different formats presented in this study, when provided with a patient’s observed score, the cutoff score, and a representation of measurement uncertainty. Although most clinicians overestimated the probability of having a threshold score of PD, average probability assessments were close to the true probability. In addition, the vast majority of decisions based on these score reports were correct. However, we should note that the large inter- and intraindividual differences found in this study indicate that interpreting measurement accuracy can be a challenging task for some clinicians. The quantile dot plot used in this study showed potential for reducing inaccuracies in probability assessments with minimal instruction. Although the average reduction of inaccuracy was modest, our results indicate that the responses were closely and evenly spread around the true probability. In addition, this increased accuracy was achieved with very minimal instruction on the use of a format that was unfamiliar to participants.

Kale et al. (2021) similarly found that quantile dot plots reduce inaccuracies in participants’ probability assessments relative to error bars. They conclude that frequency-based visualizations like quantile dot plots facilitate statistical reasoning by representing probabilities by discrete countable quantities (e.g., dots). Contrary to Kale et al., our findings did show a significant relation between inaccuracy and decision making. The difference between the findings of the two studies may be related to task familiarity: The study by Kale et al. presented a sample of a general population with a novel task, while the task in our study was designed to present a realistic and familiar scenario to participants. However, we did not find any differences in decision quality across formats. This may have been due to a relatively small difference in inaccuracy across formats, combined with the vast majority of participants correctly referring (fictional) patients to Department G. It would be interesting to explore this in future studies, by creating scenarios that favor both decisions an equal number of times.

Similar to the findings of studies by de Bruin et al. (2013) and Levontin et al. (2020), subjective understanding was not associated with inaccuracy or decision quality, in our study. Neither the SGL nor the participants’ own ranking of most understandable plots showed any relation with other outcome variables, except with each other. A main factor related to subjective understanding, as indicated by the participants’ open-ended responses, seemed to be familiarity: participants rated familiar textual and error bar formats as more understandable, despite their probability assessments being less accurate with these formats. In addition, participants reported that they associated textual formats with objectivity and accuracy, and error bars with quick and easy overviews. Since the boundaries set for any confidence interval can be arbitrarily selected, and any fixed boundary is subject to uncertainty itself, the idea that such intervals provide an objective overview is essentially false. While the additional information on the underlying distribution provided by the other formats was sometimes rated as helpful, designers of score reports should avoid overwhelming clinicians with information. As for the quantile dot plot, the number of black dots was mentioned by a few participants as particularly complex and crowding. Reducing the number of dots might make this plot seem less congested, but would also reduce the precision of the visualization.

We consider the use of a specific sample consisting of clinicians recruited from various clinical contexts as being a major strength of our study. Moreover, the sample size provided sufficient power to distinguish small differences in inaccuracy of probability assessments for the different formats. This being said, we suspect that the selective sampling may have impacted the representativeness of the study sample with respect to several characteristics: The young age group and proportion of remedial educationalists were overrepresented in the Dutch sample, which may have been the result of sampling from the professional network of the first author and the Association of Educationalists in the Netherlands, while the Norwegian sample contained a large number of psychologists with research experience and/or a PhD degree, which may have resulted from sampling from the second author’s professional network and the Norwegian Psychological Association. Although we recruited participants who were trained as mental health care practitioners, it is unknown whether all participants currently work in a mental health context. We did not suspect there would be any major influences on the conclusions, since we controlled for differences in research experience, and none of the other sample characteristics were found to significantly affect the study outcome. Likewise, although there were minor indications of selective dropout that might influence sample representativeness, differences between participants who dropped out and participants who did not were small and non-significant.

One final potential limitation is the fact that viewing conditions were not standardized. This meant that screen sizes varied from ø 6.8-inch to ø 38.8-inch monitors, and, consequently, that images were smaller for some participants than for others. We did check the influence of screen size (data not shown) and found no significant relations with both inaccuracy and decision quality. There were no other indications that screen size influenced the outcome.

By creating a realistic albeit simplified scenario for participants, we were able to operationalize the effects of different uncertainty visualizations on clinicians’ interpretations and decision quality. Although clinicians will likely consider various factors in applied settings, and not base their decision solely on a single cutoff score and expected waiting time, visualizations like the ones used in this study can provide a flexible and simple overview of test scores and their associated accuracy. The results of our study show that most clinicians were able to accurately interpret uncertainty information in different formats and base their decisions on this information. However, the same results show large interindividual differences in the way clinicians interpret the information provided in these score reports, which at times resulted in decisions that increased expected waiting time for (fictional) patients by more than a month. This study showed that plot formats such as the quantile dot plot can successfully reduce errors in interpretation and understanding, with minimal instruction.

The evidence provided in our study is sufficiently compelling to warrant wider implementation of quantile dot plots in score reports for use by trained mental health providers such as psychiatrists, psychologists, and remedial educationalists. Our research results suggest that this visualization method may have benefits in terms of facilitating correct interpretation of test scores and their associated uncertainty. Training in the interpretation of measurement accuracy and the use of quantile dot plots may help reduce the large interindividual variation in performance found in this study. Zapata-Rivera et al. (2016) showed that a brief online tutorial for teachers containing causes, definitions, illustrations, and interoperations of measurement error can improve teachers’ understanding of the method and practice. Such a tutorial may be adapted to a training program for clinical practitioners. A greater understanding of the needs and pitfalls in clinical practice may be facilitated by actively involving practitioners in the design of such a program.

As this study is one of only a handful of empirical studies on test inaccuracy reporting, and, to our knowledge, one of the first with a sample of clinical professionals, there still remain many areas worth exploring. Interesting areas for further examination include the impact of more elaborate instruction and other visualization features, such as the use of color and the use of visualizations in applied clinical contexts. In addition, there is some evidence that links clinical orientation to attitudes toward use of standardized assessment (Jensen-Doss & Hawley, 2010). Hence, clinical orientation might be a relevant user characteristic to include in further studies. Meanwhile, our results provide hopeful signs that visualizations can aid the understanding and consideration of measurement accuracy in clinical decision making. This offers an exciting new avenue for research and the development of clinical score reports and their interpretation.

## Supplemental Material

sj-docx-1-asm-10.1177_10731911221147042 – Supplemental material for Visualizing Uncertainty to Promote Clinicians’ Understanding of Measurement Error Click here for additional data file. Supplemental material, sj-docx-1-asm-10.1177_10731911221147042 for Visualizing Uncertainty to Promote Clinicians’ Understanding of Measurement Error by Niek Frans, Benjamin Hummelen, Casper J. Albers and Muirne C.S. Paap in Assessment

## Notes

1. A remedial educationalist [Dutch: *orthopedagoog*] is a behavioral scientist, who focuses on learning and behavioral disorders in children in education, youth care, and mental health care, and requires training comparable to a master’s degree in developmental psychology.

2. While the actual meaning in a frequentist framework is more nuanced (Charter & Feldt, 2001a), we chose this simple phrasing for the sake of easy communication.
