# Supplementary material for: Visualizing Uncertainty to Promote Clinicians’ Understanding of Measurement Error
Source: Assessment. 2023 Feb 1;30(8):2449–60. doi: 10.1177/10731911221147042 (PMC10623599; doi:10.1177/10731911221147042)
Supplement: sj-docx-1-asm-10.1177_10731911221147042 – Supplemental material for Visualizing Uncertainty to Promote Clinicians’ Understanding of Measurement Error [file sj-docx-1-asm-10.1177_10731911221147042.docx]

# Online supplement

| **Table S1**  *Parameter estimates of a generalized multilevel model with decision quality (1 = correct, 0 = incorrect) as the dependent variable.* | | | | | | | | | |
| --- | --- | --- | --- | --- | --- | --- | --- | --- | --- |
|  |  | Empty model | |  | Format | |  | Format + characteristics | |
|  |  | Estimate | (SE) |  | Estimate | (SE) |  | Estimate | (SE) |
| intercept |  | 1.12 | (0.107) |  | 1.16 | (0.184) |  | 1.60 | (0.210) |
| format | text (intercept) |  |  |  | - | - |  | - | - |
|  | error bar |  |  |  | –0.13 | (0.236) |  | –0.21 | (0.240) |
|  | diamond plot |  |  |  | 0.30 | (0.247) |  | 0.29 | (0.250) |
|  | quantile dotplot |  |  |  | –0.34 | (0.233) |  | –0.48 | (0.238) |
|  | violin plot |  |  |  | 0.04 | (0.239) |  | –0.01 | (0.243) |
| inaccuracy |  |  |  |  |  |  |  | –2.85 | (0.554) |
|  |  |  |  |  |  |  |  |  |  |
| level two variance $(\tau^{2})$ | | 0.847 |  |  | 0.882 |  |  | 0.857 |  |
|  |  |  |  |  |  |  |  |  |  |
| *AIC* |  | 1184.4 |  |  | 1184.8 |  |  | 1159.2 |  |
| *Note.* A logit link function was used. | | | | | | | | | |

**Explanation provided to participants with each plot type**

**Textual**: “The testscore of this patient is …, scores between … and … fall within the margin of error (95% confidence interval) for this patient.”

**Error-bar:** “The small diamond shape in the middle of the plot indicates a patient’s testscore, while the bars in the plot indicate the margin of error (95% confidence interval) for this patient.”

**Quantile dotplot:** “The small diamond shape in the middle of the plot indicates a patient’s testscore, while the twenty dots indicate the margin of error (each dot represents a probability of 5%) for this patient.”

**Violin plot:** “The small diamond shape in the middle of the plot indicates a patient’s testscore, while the area between the lines indicates the margin of error (a wider area between the lines indicates that scores are more probable) for this patient.”

**Diamond plot:** “The small diamond shape in the middle of the plot indicates a patient’s testscore, while each the surrounding diamond shapes indicate the margin of error (68% confidence-interval for the inner diamond, 95% confidence-interval for the middle diamond, and all likely scores for the outer-most diamond) for this patient.”
